# Patient Empowerment and the Exclusion of Dietary Intervention Studies. Comment on “Diet and Multiple Sclerosis: Scoping Review of Web-Based Recommendations”

**DOI:** 10.2196/17063

**Published:** 2021-02-17

**Authors:** Terry Wahls, Tyler Titcomb, Babita Bisht, Murali Ramanathan

**Affiliations:** 1 Department of Internal Medicine University of Iowa Iowa City, IA United States; 2 Department of Epidemiology University of Iowa Iowa City, IA United States; 3 Department of Pharmaceutical Sciences State University of New York Buffalo, NY United States; 4 Department of Neurology State University of New York Buffalo, NY United States

**Keywords:** multiple sclerosis, dietary interventions, clinical evidence

Beckett et al [1] performed a scoping review of online dietary recommendations for multiple sclerosis (MS) from nonscientific, internet-based webpages, available as of November 2016. The authors found 32 internet articles detailing four diets—low-fat high-fiber, low saturated fat, modified Paleolithic, and an elimination diet—and provided scientific literature for the basis of each diet. We would like to thank the authors for performing this necessary work because many people with MS seek nonconventional treatments to improve their disease outcomes. It is imperative that researchers and health care professionals understand the dietary strategies that are popular among people with MS.

However, we are concerned that the authors excluded all scientific articles regarding the outcomes of human trials using the proposed dietary strategies due to fees for obtaining these articles and the assumed lack of ability of people with MS to interpret these types of articles. In our experience, people with MS who desire to incorporate diet into their personal treatment plans are often highly informed of the scientific literature regarding dietary strategies for MS. In addition, scientific journals often are open access and/or provide lay language summaries of the article; for these reasons, we disagree with the authors' justification for excluding these articles. Furthermore, the authors conclude that online dietary advice is often the result of an individual’s experiences and has not been scientifically evaluated. However, at the time of the internet search (November 2016) performed by Beckett et al [1], the case study of Dr Terry Wahls’ personal experience [2] and results from a single-arm trial indicating that a modified Paleolithic diet is feasible [3] and associated with positive outcomes [4] had been published. Prior to the publication of the article by Beckett et al [1] in January 2019, secondary outcomes from the trial demonstrated additional improvements in progressive MS patients [5,6], and a supporting randomized controlled trial [7] was published. This is only in regard to the diet promoted by Dr Wahls and does not include the many scientific publications supporting the low-fat diet promoted by Dr Roy Swank. Finally, several online websites provide links to research articles supporting the promoted dietary strategy; thus, it is important to mention that some websites provide information on diets for MS along with relevant scientific evidence.

We agree with Beckett et al [1] that the online dietary advice for people with MS is confusing and contradictory, and that it is the responsibility of health care professionals to interpret evidence for and against online dietary advice. However, we are concerned that by excluding dietary intervention studies and not updating their search prior to publication, the authors arrived at an erroneous conclusion. Some people with MS are eager to adopt dietary changes as part of their personal treatment plan due to the limitations of conventional treatment. For this reason, we agree with the authors that the scientific community needs to rigorously evaluate dietary interventions promoted for MS such as the Swank and Wahls diets. In fact, we hope that our on-going study comparing the Wahls and Swank diets among people with relapsing-remitting MS [8] will provide some of these answers.

We would like to thank the journal editors for allowing us to reply to Beckett et al [1], who conducted an important and relevant review of the online dietary advice for people with MS. Our goal in this response is to point out that currently available scientific evidence of the clinical outcomes, limited as it may be, supports the use of dietary interventions for MS. Because people with MS often incorporate diet into their personalized treatment plans, this distinction is important to facilitate future research and support for dietary intervention studies for MS.

## Abbreviations

MS: multiple sclerosis
